# The Utility of EBUS-Guided Transbronchial Forceps Biopsy With or Without an Electrocautery Knife for the Diagnosis of Mediastinal and Hilar Lymphadenopathy

**DOI:** 10.7759/cureus.40684

**Published:** 2023-06-20

**Authors:** Vikas Pathak, Ray W Shepherd

**Affiliations:** 1 Pulmonary, Critical Care and Sleep Medicine, Virginia Institute of Lung Diseases, Richmond, USA; 2 Pulmonary and Critical Care Medicine, Virginia Commonwealth University, Richmond, USA

**Keywords:** ebus-guided forceps biopsy, mediastinal lymph nodes, lymphoma, sarcoidosis, ebus-tbna

## Abstract

Introduction: Endobronchial ultrasound-guided transbronchial needle aspiration (EBUS-TBNA) has been shown to have a high diagnostic yield for mediastinal and hilar lymph node sampling, particularly in diagnosing and staging non-small cell lung cancer. However, the diagnostic yield is lower in patients with granulomatous and lymphoproliferative disorders. We prospectively compared the feasibility, safety, and diagnostic yield of EBUS-guided lymph node forceps biopsy (EBUS-TBFB) with electrocautery knife compared to EBUS-TBNA of lymph nodes in patients with suspected granulomatous and lymphoproliferative disease.

Methods: Patients over 18 years of age with mediastinal/hilar lymph node >10 mm in size in short axis (on CT chest) who had suspected sarcoidosis/lymphoma radiologically/clinically were included in the study. Patients had EBUS-TBNA first with 21 or 22G needles which were followed by biopsy of the node with small forceps (EBUS-TBFB) through the same aspiration site to obtain samples. Electrocautery knife at 20W was used in patients where mucosal penetration was difficult, followed by passage of forceps through that site.

Results: A total of 30 patients were enrolled in the study, of which 25 patients underwent EBUS-TBFB. Eight patients had a history of lymphoma, one patient had history of squamous cell carcinoma, and one patient had history of chronic lymphocytic leukemia. A 22 gauge needle was used for aspiration in all the cases that were performed, and 1.5 mm or 1.8 mm forceps were used for the biopsy. The use of electrocautery knife at 20W (Olympus America Inc.) was required in 10/25 patients. The EC knife allowed all 10 of those to have successful entry of forceps into the lymph node. The cytology (aspiration from EBUS needle) and histopathology (from forceps) were concordant in 17 patients while it was discordant in eight patients. One patient developed pneumomediastinum after needle and forceps biopsy that required no intervention.

Conclusion: EBUS-guided forceps biopsy is a safe procedure. EC knife did successfully allow forceps entry into the lymph node in all EC knife procedures. The diagnostic yield was 73% (22/30) which improved to 86% (26/30) when both techniques were combined (TBNA and TBFB).

## Introduction

Endobronchial ultrasound-guided transbronchial needle aspiration (EBUS-TBNA) has been shown to have a high diagnostic yield for mediastinal and hilar lymph node sampling, particularly in diagnosing and staging non-small cell lung cancer [1-6]. The diagnostic evaluation of EBUS-TBNA in the evaluation of mediastinal and or hilar lymphadenopathy in conditions other than lung cancer, e.g., granulomatous diseases and lymphoproliferative diseases is less clear. Mediastinoscopy is frequently needed to establish a definite diagnosis.

The diagnostic yield of transbronchial needle aspiration in one study of 258 patients with suspected sarcoidosis was found to be 66% [7]. Similarly, in another randomized study (GRANULOMA) of 304 patients with suspected sarcoidosis, the diagnostic yield using endobronchial ultrasound and endoscopic ultrasound (EUS) was 80% [8]. Similarly, in patients with suspected lymphoproliferative disease, the sensitivity of EBUS-TBNA for diagnosis is only 57-67% de-novo or recurrent [9,10]. In comparison, the diagnostic yield of NSCLC is 96% with 100% specificity [11].

The lack of core tissue is conventionally thought to have decreased the diagnostic yield of EBUS-TBNA in patients with suspected sarcoidosis and lymphoma. We therefore prospectively compared the feasibility, safety, and diagnostic yield of EBUS-guided lymph node forceps biopsy with or without electrocautery knife compared to EBUS-TBNA needle aspiration of lymph nodes in patients with suspected granulomatous and lymphoproliferative disease.

## Materials and methods

Study setting, design, and subjects

This was a single-center prospective study, conducted from July 2014 to July 2019, undertaken at a level 1 tertiary care teaching university hospital in an urban/metropolitan city. The study was approved by the institutional review board (IRB). Patients over 18 years of age with mediastinal/hilar lymph node >10 mm in size in short axis (on CT chest) who had suspected sarcoidosis/lymphoma radiologically/clinically were included in the study.

Procedure

The procedure was performed in the endoscopy suite, under moderate sedation utilizing midazolam and fentanyl in 10 patients; while general anesthesia was used in 20 patients. All the procedures were done by interventional pulmonary team. Once the patient was adequately sedated/anesthetized, the EBUS-TBNA needle aspirations were done with a 22G needle (Olympus America Inc.). The needle was passed through the mucosal wall three to four times to obtain samples, the aspirated sample was given to the pathologist at the bedside (rapid on-site evaluation) who would review the samples for adequacy. After three to four passes, a 1.5 mm forcep (Olympus America Inc., model FB-433D) was passed through the same aspiration site. Total of two to three passes were made via forceps (Olympus America Inc., model FB-433D, 1.5 mm), and samples were obtained. If there was any difficulty in mucosal penetration with the forcep into the lymph node then we would use an electrocautery knife (ENDOCUT needle knives; Tübingen, Germany: ERBE) at 20W blended mode in a puncture technique to facilitate mucosal penetration followed by forceps biopsy through the site. It was made sure that the oxygen was reduced to 40% during the use of an electrocautery knife. EBUS-TBNA samples were sent for cytology and forceps biopsy samples were sent for surgical pathology examination. The EBUS-TBNA samples were also sent for acid-fast bacilli (AFB) smear and culture. All the patients that had EBUS-TBFB received chest X-rays prior to discharge.

Analysis

We used XLSTAT version 2014 (Denver, CO: Lumivero) to analyze continuous and non-continuous variables. Measures of central tendency and measures of dispersion were calculated for the continuous data.

## Results

A total of 30 patients were enrolled, of which 10 were done under moderate sedation and rest were done with general anesthesia. Among these 30 patients, total 50 lymph node stations were aspirated (EBUS-TBNA). However, of these 30 patients, only 25 underwent EBUS-TBFB (in three patients forceps biopsy was not performed and in two patients we were unable to pass forceps into the lymph node). Among these 25 patients, total 28 lymph node stations were biopsied using forceps. The mean age of the patients was 49 years, with 14 males and 11 females. A total of 19 Caucasian, four African American, one Asian, and one Hispanic patients participated in the study (Table 1).

**Table 1:** Demographics of the patients that participated in the study.

Total patient consented	30
Number of patients who underwent forceps biopsy	25
Sex	14 Males
	11 Females
Mean age	49 years
Race	19 Caucasian
	04 African American
	01 Asian
	01 Hispanic
Past medical history	08 Lymphoma
	01 Squamous cell carcinoma (throat)
	01 Chronic lymphocytic leukemia
Needle used	22G
Forceps size	1.5 mm +/- 1.8 mm
Electrocautery knife (20W)	10/25 patients
Cytology and Histopathology	17 concordant
	08 discordant
Morbidity	01 pneumomediastinum (no intervention required)
Mortality	None

The lymph node (LN) station most commonly biopsied were station 7 and 4R (18 and 16, respectively) while forceps biopsy was done more commonly on station 7 (16) and 4R (5) too. Another LN station used for EBUS TBNA were 2R, 2L, 10R, 11R, 11L, and 12R. The LN stations for forcep biopsy were 11L and 11R. A 22G needle was used in all the cases, and 1.5 mm or 1.8 mm forceps were used for the biopsy. The use of electrocautery knife at 20W (Olympus America Inc.) was required in 10/25 patients. The EC knife allowed all 10 of those to have a successful entry of forceps into the lymph node. The cytology (aspiration from EBUS needle) and histopathology (from forceps) were concordant in 17 patients while it was discordant in eight patients. Of these eight patients, three were found to have granuloma and one follicular lymphoma that were missed on TBNA. The three patients with granuloma were subsequently diagnosed with sarcoidosis (after ruling out secondary causes) while rest of the four patients were considered to have benign lymph nodes.

Of the 30 patients who underwent EBUS-TBNA, the diagnosis was as follows: chronic lymphocytic leukemia (CLL) (1), normal lymphoid tissue (9), small cell cancer (3), granuloma (9), melanoma (1), lymphoma (2), adenocarcinoma (1) and non-diagnostic (bronchial cells) (4). The patients who had granuloma on cytology and or histopathology were diagnosed with sarcoidosis (after secondary causes were ruled out). Of the 25 patients who underwent EBUS-TBNA and forceps biopsy, 17 patients had concordant diagnoses (cytopathology and histopathology) and eight had discordant results (Table 2). Of the eight discordant patients, in one case the TBFB provided less specific diagnostic findings than the TBNA and in three cases the TBFB provided more specific diagnostic material than the TBNA.

**Table 2:** Discordant results of the patients that participated in the study.

Cytopathology results	Histopathology results
Lymphoid elements	Acute inflammatory exudate
Granuloma	Blood and respiratory epithelium
Lymphoid elements	Follicular lymphoma
Lymphoid elements	Granuloma
Bronchial cells	Fibrous tissue
Bronchial cells	Fibrous tissue
Lymphoid elements	Fibrous tissue
Lymphoid elements	Granuloma

Of the five patients in which we were unable to pass forceps, three had granuloma and two respiratory epithelial cells. In patients where EC knives were used, five had granuloma, one melanoma, one lymphoma, one small cell cancer, and in two patients fibrous tissue. Ten patients had a history of malignancy, 8/10 had lymphoma while one had CLL and one had prior history of squamous cell carcinoma of throat (Table 3). One patient developed pneumomediastinum after needle and forceps biopsy that required no intervention.

**Table 3:** History of malignancy and outcomes.

History of malignancy	Cytopathology	Histopathology
Chronic lymphocytic leukemia	Chronic lymphocytic leukemia	Chronic lymphocytic leukemia
Squamous cell carcinoma (throat)	Small cell cancer	Small cell cancer
Lymphoma	Melanoma	Melanoma
Lymphoma	Lymphoma	Lymphoma
Lymphoma	Lymphoid elements	Granuloma
Lymphoma	Bronchial cells	Fibrous tissue
Lymphoma	Bronchial cells	Fibrous tissue
Lymphoma	Lymphoma	Lymphoma
Lymphoma	Adenocarcinoma	Adenocarcinoma
Lymphoma	Lymphoid elements	Lymphoid elements

## Discussion

Our study shows that EBUS-TBFB is a safe procedure. In majority of the patients, the same aspiration site which is used by TBNA needle can be used to insert the forceps for biopsy. Only in a few patient electrocautery knives was required. Electrocautery knife did successfully allow forceps entry into the lymph node in all EC knife procedures. We also found that TBFB did provide definitive diagnostic information beyond that of TBNA in some patients, although the study was not powered to evaluate diagnostic yield.

Gasparini et al. did a similar pilot study on 14 patients, however, compared to our study they chose the patients with LN size greater than 2 cm and included only subcarinal lymph node (station 7) [12]. Our study included lymph node >1 cm and included mediastinal and hilar lymph node stations. Out of 10 patients who used TBFB, they were able to get the diagnosis in eight of them. No complications were noted.

Chrissian et al. similarly enrolled 50 patients who underwent EBUS-TBNA followed by TBFB [13]. They compared the diagnostic yield of forceps biopsy vs. needle aspiration. Compared to our study, they had more success in sampling the hilar lymph node (36 of 74 lymph node stations samples, 49%). The overall diagnostic yield of EBUS-TBNA in 74 lymph nodes was 81% (60/74), when combined with both techniques the diagnostic yield increased to 97% (72 of 74).

Franke et al. looked at the diagnostic yield of TBFB with similar technique as ours, except that they used mini-forceps [14]. They used 21 or 22G needle followed by 0.8 mm forceps (ours were 1.5 mm forceps). They showed that the diagnostic sensitivity of TBNA increased from 50% to 82% when both procedures were combined. Similar to our case, they had one incident of pneumomediastinum which resolved without intervention.

Zhang et al. did a randomized trial to determine the diagnostic yield and safety of transbronchial mediastinal cryobiopsy vs. transbronchial needle aspiration [15]. They had 197 patients enrolled in their study with the diagnostic yield better with mediastinal cryobiopsy (91.8% vs. 79.9%) vs. needle aspiration alone. They also noted that the diagnostic yields were similar in metastatic lymphadenopathy while cryobiopsy was more sensitive in benign diseases. From safety standpoint, they had two cases of pneumothorax and one case of pneumomediastinum. This study, however, cannot be compared to our study since they did not compare the diagnostic yield of forceps biopsy vs. cryobiopsy. And although there were only three patients with complications, the learning and technical skill of using cryobiopsy is much higher than forceps biopsy. Besides, it is likely that the diagnostic yield of both procedures is the same.

It is worth mentioning that none of the above studies utilized electrocautery knife. We are the first to demonstrate the safety of electrocautery knife to facilitate the forceps into the lymph nodes. Our diagnostic yield was 73% (22/30) which improved to 86% (26/30) when both techniques were combined (TBNA and TBFB). Due to lack of availability of mini-forceps in the United States, we used 1.5 mm forceps, which was found to be safe as well. Single-center study and smaller sample size were our biggest limitations.

## Conclusions

EBUS-guided forceps biopsy is a safe procedure. Most patients do not require the use of electrocautery knife. Electrocautery knife did successfully allow forceps entry into the lymph node in all EC knife procedures. TBFB did provide definitive diagnostic information beyond that of TBNA in some patients, although the study was not powered to evaluate diagnostic yield.
